# Editorial: What have we learned over the last 25 years assessing novel food and protein allergenicity

**DOI:** 10.3389/falgy.2024.1440478

**Published:** 2024-07-18

**Authors:** Scott McClain, Gregory Ladics

**Affiliations:** ^1^ Health and Life Sciences, Statistical Analysis System Institute, Cary, NC, United States; ^2^ Product Safety, International Flavors and Fragrances, Wilmington, DE, United States

**Keywords:** food allergy, risk, safety assessment, novel, allergic potential

**Editorial on the Research Topic** What have we learned over the last 25 years assessing novel food and protein allergenicity?

Allergy science and the science of food product risk assessment has been expanding for more than 25 years. With the advent of consortia (Health and Environmental Sciences Institute; Crop Life International) dedicated to informing the public and collaborating with industry, regulatory and academia, the processes to promote safety has matured significantly. As has been noted in our session publications, the variance in allergy and the distribution of risk, generally, ranges in unknown degrees geographically. The introduction of novel foods into a population may lead to sensitization and food allergy. Although eating is a risk factor for food allergy, there are only certain foods that can cause significant food allergic reactions, with only a few of the many proteins in food accounting for such allergic reactions. What makes a few proteins allergenic, while the majority remain non-allergenic? There is still much to learn about factors influencing sensitization and the etiology of food allergy in general. Attempting to have risk review policies and processes, therefore remains a challenge to address across diverse regulatory cultures. Clearly, we are in a space now where advanced analytics, refined diagnostics, and perhaps artificial intelligence help address the new technologies of novel foods and feeds. So, what have we learned? We'll spell out some of the advances and maturation of the science and expose research gaps where work continues.

Where allergy science has moved in 25 years overlaps with the maturation of risk assessment and its origins in toxicology, more generally. In delivering regulatory science evaluations of food safety, it's been noted that “allergy assessments” really don't satisfy the needs of complex interactions across various allergens, their biological source organisms, and the human immune system response mechanisms. This is mostly due to applying an assessment framework extended from a chemical risk assessment. Today, we still do not apply a quantitative allergy assessment for novel foods and feeds. In particular, the current evolution of allergy assessment has gaps that leave out aspects that would lend towards a “true” risk assessment framework in the future. “True”, referring to considering the hazard of an allergen in combination with a quantitative understanding of the exposure and dose to elicit a clinically relevant effect.

In this Research Topic, Fernandez et al., identify research gaps and future needs for allergy safety and allergen prediction in food safety. With significant advances in biotechnology and the need for developing alternative food protein sources, the authors advocate updating safety assessment prediction approaches for food allergy. They suggest a focus on clinical relevance and the development of a fit-for-purpose database for specific risk assessment goals for global foods of the future. Krutz et al. highlight the inclusion of key aspects of learning how to apply safety assessment methods and the risk paradigms of toxicology to allergology. One aspect is inclusion of open evaluations of expected exposure to any novel or transferred proteins (by way of genetic modification). Allergy, from a clinical standpoint, typically relies on IgE immunoglobulin as a diagnostic and response metric. The authors remind us that tracking and classifying clinical risk is limited when applied in isolation within the context of risk assessments. The research of Fernandez et al., also offers also offers the undeniable global perspective of food safety; they identified several interesting points regarding food allergy in Columbia. In particular, the region of Columbia differs from others in the observation of rate and expected prominent allergens. A high rate of consumption for a food group (fruit) does not necessarily result in a high rate of allergy; this may likely be proof of an obvious physiological phenomenon of repeated exposure (early and often) that results in tolerance and suppressed clinical relevance in the population. In the end, locality defined at various level of geographical scale are a feature that also vary for each allergen, independently. A complex variable in clinical allergy that requires much more study.

Currently, no single factor is recognized as a predictor for protein allergenicity. Therefore, a weight-of-the-evidence approach, which considers a variety of factors and approaches for an overall assessment of allergenic potential, will need to continue. To date, there is an inability to replicate the physiological processes of allergy sensitization, elicitation and tolerance in easy-to-apply bench tests for novel foods. Nevertheless, over the last 25 years there has been progress on developing new techniques to better predict the potential of a novel protein to produce food allergy. Such areas include the development of *in silico*, machine learning approaches. These algorithmic programs attempt to help predict the *de novo* allergenic potential of a novel protein(s). Advances in the quality of whole genome sequencing and advanced bioinformatic tools are helping more accurately predict potential allergenic protein cross-reactivity among structurally similar proteins. With the advent of artificial intelligence, the prediction of the potential allergenicity of novel protein(s) will become even more refined increasing our confidence in food allergy risk assessments.

